# Comparative Study of Young and Mature *Dendropanax morbifera* Leaves: Superior Neuroprotective Efficacy of Young Leaves Through Enhanced Anti-Inflammatory and Metabolic Modulation

**DOI:** 10.3390/plants15132056

**Published:** 2026-07-02

**Authors:** Da-un Jung, Ahreum Lee, Dalnim Kim, Hyun-Jeong Yang

**Affiliations:** 1 Jeju Dental Clinic for Persons with Special Needs, Jeju National University Hospital, Jeju 63241, Republic of Korea; jung_dental@naver.com; 2 Korea Institute of Brain Science, Seoul 06022, Republic of Korea; dkfma5025@gmail.com (A.L.); hoipig0326@gmail.com (D.K.); 3 Department of Integrative Healthcare, University of Brain Education, Cheonan 31228, Republic of Korea

**Keywords:** *Dendropanax morbifera*, leaf age, chlorogenic acid, neuroinflammation, microglia, BDNF

## Abstract

Neuroinflammation, driven by microglial activation and oxidative stress, is a key pathological feature of various neurodegenerative diseases. *Dendropanax morbifera* Léveille (DM) is a medicinal plant known for its diverse pharmacological activities; however, the influence of leaf developmental stage on its neuroprotective potential remains poorly understood. In this study, we compared the phytochemical profiles of young DM (YDM) and mature DM leaves and evaluated their effects on neuronal metabolism and microglia-mediated neuroinflammation. HPLC analysis revealed that YDM contained approximately 2.4-fold higher levels of chlorogenic acid than DM, while DM exhibited higher quercetin content. In differentiated N2A neuronal cells, YDM treatment significantly upregulated the expression of key metabolic and mitochondrial regulators, including PGC-1α, PPARγ, and CPT2, suggesting enhanced mitochondrial and metabolic regulatory signaling related to biogenesis and fatty acid β-oxidation. Under inflammatory conditions, YDM more potently suppressed the secretion of pro-inflammatory cytokines (IL-6 and TNF-α) in LPS-stimulated BV2 microglia compared to DM. Furthermore, in N2A cells treated with BV2-conditioned medium, both extracts effectively mitigated reactive oxygen species production and restored brain-derived neurotrophic factor expression. These findings demonstrate that leaf age is a critical determinant of the phytochemical composition and biological activity of DM. Our results suggest that chlorogenic acid-rich YDM preparations may offer superior therapeutic advantages in targeting neuroinflammatory and metabolic dysregulation in the central nervous system.

## 1. Introduction

Neuroinflammation is a hallmark feature of various neurodegenerative and neuropsychiatric disorders, including Alzheimer’s disease, Parkinson’s disease, and major depressive disorder, and is closely associated with microglial activation and oxidative stress [1,2]. Once activated, microglia secrete inflammatory cytokines such as interleukin-6 (IL-6) and tumor necrosis factor-α (TNF-α). These mediators impair neuronal mitochondrial function and suppress brain-derived neurotrophic factor (BDNF) signaling—a critical factor for neuroprotection—ultimately leading to reduced neuronal survival and synaptic plasticity [1,2,3].

Accumulating evidence suggests that preserving metabolic homeostasis, mitochondrial biogenesis, and antioxidant defense systems in neurons is crucial for maintaining resilience against microglia-derived neuroinflammatory damage [4]. Key regulators of these processes include AMP-activated protein kinase (AMPK), sirtuin 1 (SIRT1), and peroxisome proliferator-activated receptor gamma coactivator 1-alpha (PGC-1α), which coordinately regulate mitochondrial biogenesis and fatty acid oxidation. In particular, PGC-1α acts as a coactivator for the transcription factors peroxisome proliferator-activated receptor (PPAR) α and PPARγ, inducing the expression of mitochondrial fatty acid β-oxidation enzymes such as carnitine palmitoyltransferase (CPT) 1A and CPT2. This induction promotes the oxidation of long-chain fatty acids and energy production, thereby contributing to the restoration of cellular metabolic homeostasis. Furthermore, nuclear factor erythroid 2-related factor 2 (NRF2), recognized as the master regulator of the antioxidant response, interacts with these pathways to simultaneously enhance mitochondrial function and antioxidant defenses [5,6,7].

*Dendropanax morbifera* Léveille (DM), an evergreen tree belonging to the family *Araliaceae*, is an endemic species native to the Republic of Korea and has been utilized traditionally as a medicinal plant. Various in vitro and in vivo studies have reported that DM leaf extracts exhibit a broad spectrum of pharmacological activities, including antioxidant, anti-inflammatory, and neuroprotective effects [8,9,10].

Phytochemical analyses have shown that DM leaves contain multiple phenolic constituents, among which chlorogenic acid, rutin, and quercetin are representative bioactive compounds. Previous studies have reported that the contents of chlorogenic acid, rutin, and quercetin in DM leaf extracts vary depending on harvest time, cultivation period, and extraction solvent, ranging from 9.13 to 36.55, 27.32 to 116.71, and 2.27 to 20.14 mg/g extract, respectively [11,12,13]. Notably, higher contents of chlorogenic acid and rutin have been correlated with enhanced antioxidant and radical scavenging activities [11,14].

Chlorogenic acid is one of the predominant phenolic acids in DM leaves and has been widely reported to exert anti-inflammatory and neuroprotective effects across various experimental models. These effects are achieved by inhibiting microglial activation, reducing the secretion of pro-inflammatory cytokines, and mitigating oxidative stress [11,14,15]. In both microglia and brain tissues, chlorogenic acid has been shown to suppress the lipopolysaccharide (LPS)-induced production of nitric oxide (NO) and cytokines, while also ameliorating neurobehavioral deficits in rodent models of neuroinflammation and neurotoxicity [16,17]. Furthermore, chlorogenic acid is reported to regulate mitochondrial function and energy metabolism [18], suggesting that its neuroprotective efficacy may be linked to these mechanisms.

However, little is known regarding how the concentrations of these key bioactive compounds differ between young and mature leaves of DM. Furthermore, it remains largely unexplored whether such variations lead to distinct neuroprotective profiles under conditions of microglia-mediated neuroinflammation. Although the developmental stage of a plant is a critical determinant of its secondary metabolite composition, the influence of leaf age on the bioactivity of DM has received little attention. Given that many medicinal plants exhibit significant fluctuations in their chemical profiles according to leaf age [19,20,21], it is highly probable that young DM (YDM) and mature DM leaves possess distinct concentrations of key bioactive compounds. Consequently, such variations may lead to differential levels of anti-inflammatory and neuroprotective activities.

In the present study, we focused on chlorogenic acid, rutin, and quercetin—the primary bioactive constituents reported in DM—to compare their concentrations in young and mature DM leaf extracts. We further evaluated their effects on neuronal metabolism and the expression of antioxidant-related proteins under physiological conditions, as well as their neuroprotective efficacy within a microglia-mediated neuroinflammatory environment.

Specifically, we investigated the impact of YDM and DM on metabolic and antioxidant protein expression in differentiated N2A cells. Furthermore, we assessed their ability to suppress LPS-induced NO and pro-inflammatory cytokine secretion in BV2 microglia. Finally, using a BV2 microglia-conditioned medium model designed to mimic microglia-derived neuroinflammatory states, we evaluated the effects of these extracts on reactive oxygen species (ROS) production and BDNF expression.

## 2. Results

### 2.1. Young Dendropanax morbifera Leaf Extracts Show a Higher Concentration of Chlorogenic Acid Compared to Mature Leaf Extracts

**Figure 1:**
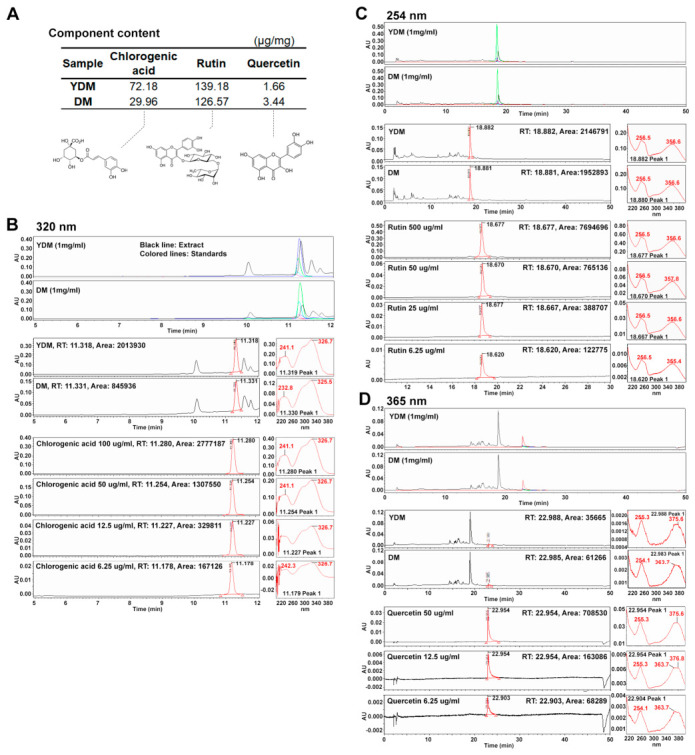
HPLC analysis of three major components in young and mature *Dendropanax morbifera* leaf extracts. (**A**) Contents of chlorogenic acid, rutin, and quercetin in young and mature *Dendropanax morbifera* leaf extracts (YDM and DM, respectively). The unit μg/mg represents the phytochemical content per milligram of freeze-dried leaf extract. (**B**–**D**) HPLC chromatograms of YDM and DM extracts (1 mg/mL) and their respective standards detected at specific wavelengths. The black lines represent the extracts, and the colored lines represent the standards at varying concentrations. (**B**) Chlorogenic acid (320 nm): (top) standards at 100 (blue), 50 (dark green), 12.5 (light green), and 6.25 (red) μg/mL; (bottom) 100 (light green), 50 (dark green), 12.5 (red), and 6.25 (brown) μg/mL. (**C**) Rutin (254 nm): standards at 500 (green), 50 (blue), 25 (red), and 6.25 (light blue) μg/mL. (**D**) Quercetin (365 nm): standards at 50 (red), 12.5 (blue), and 6.25 (green) μg/mL.

Previous studies have reported that chlorogenic acid, rutin, and quercetin are the major constituents of DM leaves [11,13]. To investigate whether there are differences in these key components between YDM and DM, high-performance liquid chromatography (HPLC) analysis was performed (Figure 1). Both YDM and DM contained all three compounds; however, their concentrations differed. The chlorogenic acid content was approximately 2.4-fold higher in YDM than in DM (72.18 vs. 29.96 μg/mg), while rutin levels were comparable between the two groups (139.18 vs. 126.57 μg/mg). In contrast, quercetin was approximately 2-fold higher in DM than in YDM (3.44 vs. 1.66 μg/mg). Chlorogenic acid has been reported to attenuate neuroinflammation by reducing microglial activation and the release of pro-inflammatory cytokines in various models [15,17,22]. Therefore, in this study, we evaluated whether YDM, which is richer in chlorogenic acid, would exert a greater anti-neuroinflammatory effect than DM.

### 2.2. Young Dendropanax morbifera Leaf Extracts Enhance the Expression of Metabolic and Mitochondrial-Related Factors in Neuronal Cells

Under neuroinflammatory conditions, neuronal energy metabolism and mitochondrial function play critical roles in neuronal protection. In addition, activation of antioxidant defense systems is essential for alleviating oxidative stress induced by neuroinflammation [23]. Accordingly, using differentiated N2A cells as a model, we investigated the effects of YDM and DM on the expression of proteins related to metabolism and antioxidant responses in neuronal cells.

**Figure 2:**
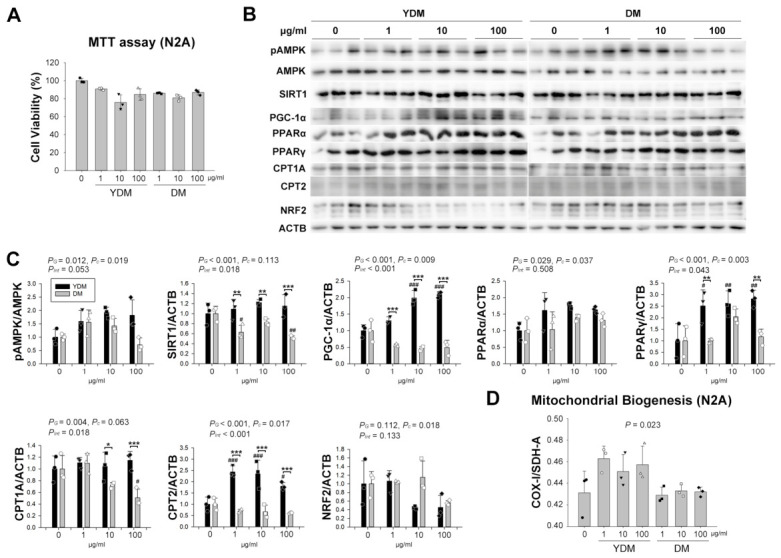
Enhanced expression of metabolism-related proteins by young *Dendropanax morbifera* leaf extracts compared to mature leaf extracts in differentiated neuronal N2A cells. (**A**) Cell viability (%) of N2A cells treated with YDM or DM at the indicated concentrations for 24 h, determined by MTT assay. (**B**) Western blot analysis of factors related to upstream metabolic regulators (p-AMPK, SIRT1, PGC-1α), mitochondrial fatty acid oxidation (PPARα, PPARγ, CPT1A, CPT2), and antioxidant response (NRF2) in differentiated N2A cells treated with YDM or DM for 48 h. (**C**) Quantitative analysis of the Western blot results shown in (**B**). (**D**) Mitochondrial biogenesis. *N* = 3 independent cultures. Data were analyzed using two-way ANOVA followed by the Holm–Sidak method for multiple comparisons (**C**) and one-way ANOVA (**D**). *P_G_*, *P_C_*, and *P_INT_* represent the *p* values for group, concentration, and their interaction, respectively. For comparisons between groups at each concentration: *, *p* < 0.05; **, *p* < 0.01; ***, *p* < 0.001. For comparisons between the control (0 μg/mL) and other concentrations: #, *p* < 0.05; ##, *p* < 0.01; ###, *p* < 0.001. The unit μg/mL refers to the final concentration of the freeze-dried extract diluted in the cell culture medium. Bars indicate mean ± SD. Different symbols in the bar graph represent distinct groups.

Cell viability remained above approximately 80% following a 24 h treatment with YDM and DM at concentrations of 1, 10, and 100 μg/mL of freeze-dried extract in the cell culture medium (Figure 2A). Based on these results, differentiated N2A cells were treated with these three concentrations, and changes in the expression of metabolism-related factors were analyzed by Western blotting (Figure 2B,C). The following markers were examined: upstream metabolic regulators (p-AMPK, SIRT1, PGC-1α), mitochondrial fatty acid oxidation (PPARα, PPARγ, CPT1A, CPT2), and antioxidant response (NRF2).

While DM treatment had no effect on PGC-1α, PPARγ, and CPT2, YDM treatment significantly increased their expression; specifically, PPARγ and CPT2 were upregulated from 1 μg/mL, whereas PGC-1α showed significant induction starting at 10 μg/mL. Although a significant main effect of YDM treatment was observed for p-AMPK/AMPK and PPARα compared to DM (*p* < 0.05), post hoc analysis revealed no statistically significant differences between specific groups. NRF2 expression did not differ between the two extracts. In contrast, SIRT1 and CPT1A expression decreased following DM treatment, resulting in significantly higher levels in the YDM group compared to DM (Figure 2C).

PGC-1α is a master regulator of mitochondrial biogenesis and is known to promote mitochondrial biogenesis and oxidative metabolism through interaction with PPARγ. CPT2, located in the inner mitochondrial membrane, is a key enzyme regulating the β-oxidation of long-chain fatty acids [5,23,24,25,26]. Therefore, the upregulation of these proteins by YDM suggests a potential enhancement of mitochondrial biogenesis-related regulatory signaling. To further examine this possibility, a mitochondrial biogenesis assay was performed, which revealed a significant difference among groups by one-way analysis of variance (ANOVA) (*p* = 0.023). Notably, YDM showed slightly higher mean values than DM at all concentrations, although the magnitude of the change was modest (Figure 2D).

### 2.3. Young Dendropanax morbifera Leaf Extracts Suppress LPS-Induced Pro-Inflammatory Cytokine Production More Strongly than Mature Leaf Extracts in BV2 Cells

Next, we evaluated whether YDM more effectively attenuates microglia-mediated neuroinflammation compared to DM. BV2 cells were pre-incubated with YDM or DM, followed by LPS stimulation to assess nitrite levels and the secretion of pro-inflammatory cytokines (IL-6 and TNF-α). Subsequently, the conditioned media were applied to differentiated N2A cells to examine changes in ROS production.

**Figure 3:**
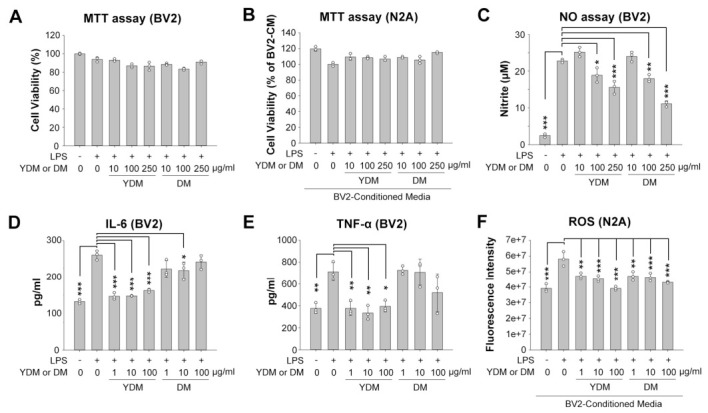
Alleviating effects of young versus mature *Dendropanax morbifera* leaf extracts on neuroinflammation-mediated neurotoxicity. BV2 cells were pre-incubated with vehicle, YDM or DM for 1 h, then stimulated with LPS (final concentration: 100 ng/mL) for 24 h (**A**,**C**) or 72 h (**D**,**E**). The resulting supernatant (conditioned media, CM) was used for the indicated assays (**C**–**E**) and for treating differentiated N2A cells for an additional 24 h (**B**,**F**). (**A**) Cell viability (%) of BV2 cells treated with YDM or DM at the indicated concentrations for 24 h. (**B**) Cell viability (%) of N2A cells treated with BV2-CM. (**C**) Nitrite levels (μM) in the supernatant of BV2 cells. (**D**) IL-6 and (**E**) TNF-α levels (pg/mL) in the supernatant of BV2 cells. (**F**) ROS levels in N2A cells treated with BV2-CM. *N* = 3 independent cultures. Data were analyzed using one-way ANOVA followed by the Holm–Sidak method. For comparisons between LPS-only and other conditions: *, *p* < 0.05; **, *p* < 0.01; ***, *p* < 0.001. Bars indicate mean ± SD. The circles represent the individual data points from independent experiments.

Cell viability was maintained above approximately 80% at concentrations up to 250 μg/mL in both BV2 and N2A cells (Figure 3A,B). Based on these viability results, concentrations up to 250 μg/mL were used for the subsequent inflammatory assays in BV2 cells. In the NO assay, both YDM and DM reduced LPS-induced nitrite production in a dose-dependent manner (Figure 3C). Notably, analysis of representative pro-inflammatory cytokines revealed that YDM more markedly suppressed LPS-induced IL-6 and TNF-α secretion compared to DM (Figure 3D,E).

In experiments measuring ROS levels in N2A cells treated with BV2-conditioned media, both YDM and DM dose-dependently attenuated ROS production (Figure 3F). Taken together, YDM more effectively inhibited pro-inflammatory cytokine secretion than DM, while both extracts shared a common antioxidant protective effect against microglia-derived oxidative stress.

### 2.4. Young Dendropanax morbifera Leaf Extracts More Effectively Restore LPS-Induced Reductions in BDNF

In neuroinflammatory conditions, excessive microglial activation impairs BDNF signaling [27,28]. As shown earlier, under physiological conditions, YDM increased PGC-1α and PPARγ expression in N2A cells and showed differential effects on CPT1A expression compared to DM (Figure 2C). Based on this, we evaluated the effects of YDM and DM on BDNF and metabolism-related protein expression under pathological conditions involving microglia–neuron interactions.

**Figure 4:**
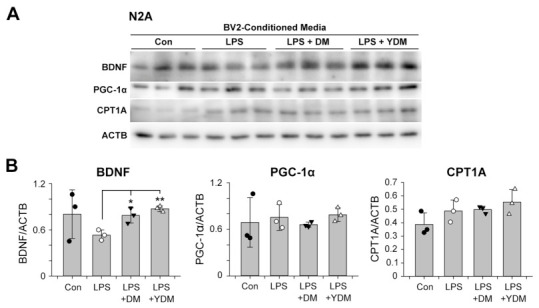
Recovery of BDNF protein expression by young and mature *Dendropanax morbifera* leaf extracts. BV2 cells were pre-incubated with vehicle, YDM, or DM for 1 h, then stimulated with LPS (100 ng/mL) for 72 h. N2A cells were treated with the BV2-conditioned media for 24 h, and then collected for protein analysis. (**A**) Representative Western blot images of the indicated proteins in N2A cells. (**B**) Densitometric quantification of the Western blots shown in (**A**). The concentration of DM and YDM used was 100 μg/mL. *N* = 3 independent cultures. Data were analyzed using Student’s *t*-test. *, *p* < 0.05; **, *p* < 0.01. Bars indicate mean ± SD. Different symbols in the bar graph represent distinct groups.

BV2 cells were pretreated with YDM or DM, followed by LPS stimulation, and the resulting conditioned media were applied to differentiated N2A cells to assess changes in protein expression of BDNF, PGC-1α, and CPT1A (Figure 4A,B). LPS treatment slightly reduced the mean level of BDNF expression in N2A cells, which was significantly restored by both DM and YDM. Although the recovery was slightly greater with YDM than with DM, the difference was not statistically significant. In addition, no significant changes were observed in PGC-1α or CPT1A expression under these conditions (Figure 4B).

## 3. Discussion

**Figure 5:**
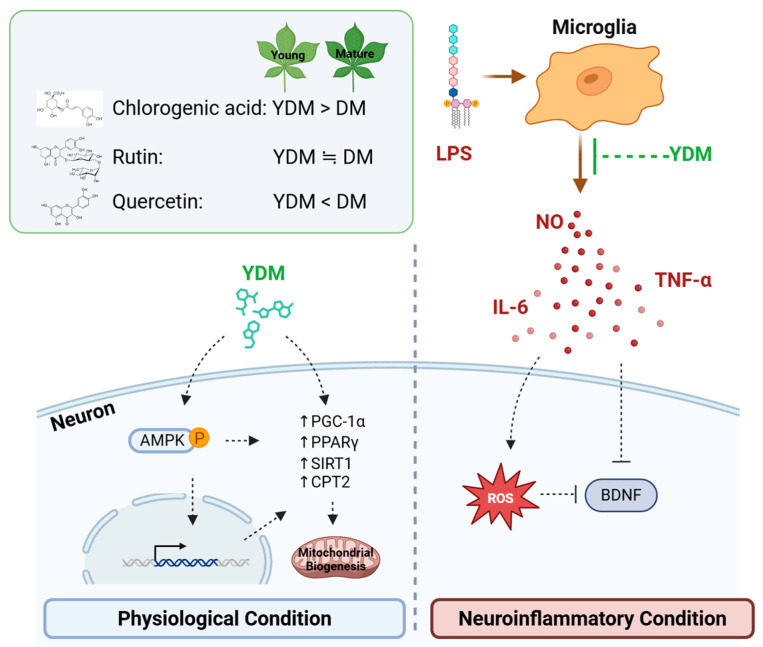
Proposed schematic model of the in vitro neuroprotective effects of young *Dendropanax morbifera* leaf extract (YDM) compared with mature leaf extract (DM). YDM contains a higher level of chlorogenic acid than DM and is associated with enhanced metabolic and mitochondrial regulatory signaling in differentiated N2A neuronal cells. In parallel, YDM suppresses LPS-induced inflammatory responses in BV2 microglia, leading to reduced secretion of IL-6 and TNF-α. Under microglia-conditioned neuroinflammatory conditions, YDM attenuates ROS accumulation and restores BDNF expression in N2A cells. Overall, YDM exhibits comparatively stronger in vitro anti-inflammatory and neuroprotective effects than DM. The dashed arrow indicates the putative pathway hypothesized in this study. The brown-colored text represents LPS and the LPS-induced inflammatory factors.

The present study demonstrates that YDM, which contains more chlorogenic acid than DM, exerts stronger in vitro protective effects against neuroinflammation. Compared to DM, YDM more potently upregulated the expression of proteins related to mitochondrial biogenesis and fatty acid oxidation in neuronal cells and more effectively suppressed LPS-induced pro-inflammatory cytokine secretion in microglia. Conversely, both extracts exhibited comparable efficacy in inhibiting NO production in BV2 cells and in restoring ROS levels and BDNF expression in differentiated N2A cells challenged with LPS-stimulated BV2-conditioned media (Figure 5).

These findings suggest that the age-dependent variations in the phytochemical composition of DM leaves are functionally significant. Furthermore, YDM showed comparatively stronger in vitro effects than DM under the present experimental conditions, suggesting that it may have greater potential for modulating neuroinflammatory pathways. Further mechanistic studies and in vivo validation will be required to determine whether these in vitro differences translate into meaningful therapeutic advantages.

Our phytochemical analysis revealed that, although both YDM and DM contained chlorogenic acid, rutin, and quercetin, their quantitative distribution patterns differed significantly. In particular, chlorogenic acid was more abundant in YDM than in DM. In the present study, the chlorogenic acid content was 72.18 μg/mg of freeze-dried extract in YDM and 29.96 μg/mg of freeze-dried extract in DM, indicating an approximately 2.4-fold higher content in YDM (Figure 1A). Notably, Eom et al. reported a chlorogenic acid content of 34.33 ± 0.16 μg/mg extract in DM leaf extract under optimal extraction conditions, indicating that the chlorogenic acid level observed in YDM was higher than this previously reported benchmark, whereas the level in DM was comparable [12]. When converted to a leaf dry matter basis using the extraction yields of the freeze-dried extracts, the chlorogenic acid content corresponded to 10.11 mg/g leaf dry matter for YDM and 3.60 mg/g leaf dry matter for DM. Although these values are lower than those reported for coffee leaves (19.208–80.836 mg/g leaf dry matter) [29], YDM still exhibited a 2.8-fold higher chlorogenic acid level than DM on a dry matter basis. This pattern is consistent with that reported for coffee leaves, in which young leaves contained, on average, approximately two-fold higher chlorogenic acid levels than mature leaves, with interspecific variation ranging from 1.4- to 3.8-fold [29]. Similarly, in the medicinal plant *Vaccinium dunalianum*, chlorogenic acid levels were also found to be higher in younger leaves, a phenomenon attributed to the upregulation of genes involved in chlorogenic acid biosynthesis during early leaf development [30]. Taken together, these findings suggest that leaf developmental stage is an important factor influencing chlorogenic acid accumulation in *Dendropanax morbifera*, as has also been observed in certain other plant species.

Chlorogenic acid has been repeatedly identified as a key mediator of anti-inflammatory and neuroprotective activities, including the inhibition of microglial activation, reduction in NO and pro-inflammatory cytokines, and mitigation of oxidative damage in neurons [11,14,15,16,17]. Consequently, the elevated chlorogenic acid content in YDM may be associated, at least in part, with the enhanced biological activities observed in this study, including the stronger suppression of cytokine secretion and the increased expression of metabolism-related proteins in neurons. In contrast, the higher quercetin content in DM did not translate into similar potent effects under the present experimental conditions.

At the neuronal level, unlike DM, YDM significantly increased the expression of PGC-1α, PPARγ, and CPT2 across most concentrations tested in differentiated N2A cells. This suggests that YDM may influence signaling pathways associated with mitochondrial biogenesis and mitochondrial fatty acid β-oxidation. PGC-1α is a well-recognized master regulator that modulates mitochondrial biogenesis and oxidative metabolism, acting in part through interactions with nuclear receptors such as PPARγ. Furthermore, CPT2, localized in the inner mitochondrial membrane, plays a critical role in the β-oxidation of long-chain fatty acids [23,31]. Given the simultaneous upregulation of these proteins, together with the modest increase observed in the mitochondrial biogenesis assay, YDM may support mitochondrial regulatory adaptation in neurons. However, because direct functional indices such as ATP production or mitochondrial membrane potential were not measured, the present data should not be interpreted as definitive evidence of improved mitochondrial function. Such metabolic reinforcement is particularly crucial in neuroinflammatory contexts, where mitochondrial dysfunction and impaired ATP production contribute to neuronal vulnerability.

Interestingly, while the p-AMPK/AMPK ratio and PPARα levels tended to be higher in the YDM-treated group compared to the DM group, these differences did not reach statistical significance, and NRF2 levels remained comparable between the two. In contrast, the expression of SIRT1 and CPT1A was significantly reduced following DM treatment, resulting in a marked disparity between YDM and DM. Given that AMPK, SIRT1, and PGC-1α constitute a core signaling axis that promotes mitochondrial biogenesis and oxidative metabolism [5], the preservation of SIRT1 and the upregulation of PGC-1α by YDM may be particularly vital for maintaining neuronal energy homeostasis under stressful conditions. Collectively, these data suggest that YDM actively enhances key components of the mitochondrial biogenesis and fatty acid oxidation pathways.

Regarding microglial activation, both YDM and DM reduced LPS-induced nitrite production in BV2 cells in a concentration-dependent manner, confirming that both extracts possess anti-inflammatory properties consistent with previous reports on DM leaf preparations [9,10]. Notably, however, YDM exerted a more potent inhibitory effect on the secretion of IL-6 and TNF-α than DM, highlighting a qualitative difference between the two extracts in modulating inflammatory cytokine responses. Importantly, the stronger suppression of IL-6 and TNF-α by YDM was not accompanied by a corresponding reduction in BV2 cell viability, as DM showed similar or lower viability at some concentrations but weaker cytokine inhibition. This pattern suggests that the observed anti-inflammatory effects are unlikely to be explained solely by nonspecific cytotoxicity. In addition, the same treatment range increased mitochondrial biogenesis-related signaling and metabolic regulator expression in differentiated N2A cells, rather than simply suppressing cellular activity. Given that IL-6 and TNF-α are key mediators of microglia-mediated neurotoxicity [32,33], the superior cytokine-suppressing efficacy of YDM may serve as a central mechanism underlying its enhanced neuroprotective profile.

BDNF plays a central role in neuronal survival, synaptic plasticity, and cognitive function, and its suppression via microglial activation is a hallmark of neuroinflammatory states [34]. Previous studies have demonstrated that DM leaf extracts exert beneficial effects on neurotrophic pathways in vivo, alleviating mercury-induced spatial memory impairment and reduced hippocampal neurogenesis [35]. Our findings extend these prior reports by showing that both quercetin-rich DM and chlorogenic acid-rich YDM preparations can reverse inflammation-induced neuronal BDNF depletion, suggesting potential modulation of microglial-mediated pathways. Although PGC-1α and CPT1A levels did not change significantly under BV2-conditioned media conditions, the basal enhancement of metabolic and mitochondrial-related proteins by YDM under physiological conditions suggests that it may provide a metabolic buffer. This baseline fortification likely supports BDNF-dependent neuroplasticity during inflammatory challenges.

Several limitations of the present study warrant consideration. First, because this research was conducted using immortalized cell lines (BV2 and N2A), it only partially reflects the intricate complexity of primary microglia–neuron interactions in vivo. Second, our analysis focused on a limited scope of phytochemicals (chlorogenic acid, rutin, and quercetin); since other individual constituents were not systematically quantified or manipulated, it is difficult to definitively attribute the observed effects to a single specific compound. Nonetheless, the association between elevated chlorogenic acid content and enhanced anti-inflammatory and mitochondrial regulatory effects suggests that chlorogenic acid may contribute to the superior activity of YDM. Because the present study compared only two leaf-age groups, this relationship should be interpreted as associative rather than causal. Future studies including multiple developmental stages and a broader range of chlorogenic acid levels will be required to better define the relationship between leaf age, phytochemical composition, and biological activity. Third, the mitochondrial biogenesis assay showed only a modest change, and direct functional indicators of mitochondrial activity, such as ATP production, mitochondrial membrane potential, or oxygen consumption, were not evaluated. Accordingly, the present findings should be interpreted as evidence of altered mitochondrial regulatory signaling rather than definitive enhancement of mitochondrial function.

In conclusion, our data demonstrate that YDM, which is enriched in chlorogenic acid relative to DM, offers distinct advantages in promoting the expression of metabolic and mitochondrial-related proteins in neurons under physiological conditions. Furthermore, YDM significantly suppresses microglial pro-inflammatory cytokine production under LPS-induced inflammatory conditions. These findings emphasize that leaf age is a critical factor in determining the neuroprotective potential of DM. Our results suggest that chlorogenic acid-rich YDM preparations warrant further investigation as plant-derived materials with potential relevance to neuroinflammation and neurotrophic dysfunction.

## 4. Materials and Methods

### 4.1. Plant Materials and Extraction

The young and mature leaves of *Dendropanax morbifera* (DM) were collected in June 2022 from 9-year-old trees cultivated in Buhwang-ri, Bogil-myeon, Wando-gun, Republic of Korea. The plant material was identified by Mr. In-Joo Lee of Buyong Hwangchil Farm, from whom the material was also obtained. The voucher specimen was deposited at the Warm-Temperate and Subtropical Forest Research Center under resource management number WFRC 10034298 and collection number 71531. The deposition was made by Prof. Da-un Jung of Jeju National University. The harvested leaves were air-dried and subsequently extracted with distilled water at 80 °C for 8 h. After cooling naturally to room temperature, the extracts were filtered and freeze-dried for further analysis. The extraction yields, calculated as the ratio of freeze-dried extract weight to leaf dry weight, were 14% and 12% for YDM and DM, respectively.

### 4.2. HPLC Analysis

Quantitative analysis of bioactive compounds was performed using an HPLC system equipped with a UV detector. The analytical conditions were based on our previously reported method [36]. Specifically, chromatographic separation was carried out on a Kromasil 100-3.5-C18 column (4.6 × 250 mm, 5 μm particle size). The flow rate was maintained at 1 mL/min, and the injection volume was set at 10 μL for all analyses. The column temperature was kept at room temperature. The mobile phase composition and detection wavelengths were optimized for each compound as follows.

For chlorogenic acid, the mobile phase consisted of (A) acetonitrile and (B) 1% acetic acid in water. The gradient elution program was as follows: 0–5 min, 0–10% A; 5–10 min, 10–25% A; 10–15 min, 25–65% A; and 15–20 min, 65–100% A. Detection was performed at 320 nm. For quercetin and rutin, the mobile phase consisted of (A) methanol and (B) water, with the following gradient program: 0–20 min, 10–65% A; 20–40 min, 65–100% A; 40–45 min, 100% A; 45–47 min, 100–10% A; and 47–50 min, 10% A. Detection wavelengths were set at 365 nm for quercetin and 254 nm for rutin.

### 4.3. Cell Culture and Treatment

The murine microglial cell line BV2 and the neuroblastoma cell line N2A were obtained from the long-term laboratory stock of the University of Brain Education and maintained in our repository. For identity verification, the cell lines were referenced by their Cellosaurus accessions (RRID:CVCL_0182 for BV2 and RRID:CVCL_0470 for N2A). Cells were maintained in Dulbecco’s Modified Eagle Medium (DMEM) supplemented with 10% heat-inactivated fetal bovine serum (FBS) and 1% penicillin-streptomycin at 37 °C in a humidified atmosphere containing 5% CO_2_. Cells were confirmed to be mycoplasma-free prior to use. For differentiation, N2A cells were cultured in DMEM supplemented with 1% FBS and 20 μM retinoic acid.

Freeze-dried extracts were reconstituted in distilled water immediately before use. An equivalent volume of distilled water was added to the control cells to exclude solvent-related effects.

To induce inflammation, BV2 cells were pretreated with YDM, DM, or vehicle for 1 h, followed by stimulation with 100 ng/mL LPS for 72 h. The culture supernatants were collected for the measurement of IL-6 and TNF-α levels. For the neurotoxicity model, N2A cells were treated with BV2-conditioned medium for an additional 24 h to evaluate neuroinflammation-mediated toxicity and to collect samples for Western blot analysis.

### 4.4. Measurement of Inflammatory Cytokines, NO, Cell Viability, and ROS

The levels of secreted TNF-α and IL-6 in culture supernatants were quantified using mouse DuoSet ELISA kits (R&D Systems, Minneapolis, MN, USA) according to the manufacturer’s instructions. NO production was measured using the Griess reaction. Equal volumes of conditioned medium and Griess reagent (composed of 1% sulfanilamide in 5% phosphoric acid and 0.1% N-1-naphthylethylenediamine dihydrochloride, diluted 1:1 with distilled water) were mixed and incubated. The absorbance was then measured at 570 nm using a microplate reader (VICTOR Nivo; PerkinElmer, Waltham, MA, USA). Cell viability was evaluated using an MTT-based assay according to the manufacturer’s instructions (EZ-Cytox kit, DogenBio, Seoul, Republic of Korea). Intracellular ROS levels were measured using a ROS assay kit (ab113851, Abcam, Cambridge, UK) according to the manufacturer’s protocol. Mitochondrial biogenesis was assessed using a commercial assay kit (ab110217, Abcam, Cambridge, UK) following the manufacturer’s instructions.

### 4.5. Western Blot Analysis

Cells were lysed on ice using chilled RIPA buffer (WSE-7420, ATTO, DAWINBIO Inc., Hanam, Republic of Korea), followed by centrifugation at 15,000 rpm for 15 min at 4 °C. The resulting supernatant was collected and protein concentrations were quantified. Equal amounts of protein were mixed with sample buffer, denatured by boiling, and separated via SDS-PAGE before being transferred onto PVDF membranes. The membranes were blocked with EZBlock Chemi (AE-1475, ATTO, Tokyo, Japan) for 30 min at room temperature and subsequently incubated with specific primary antibodies overnight at 4 °C. After thorough washing, the membranes were incubated with secondary antibodies for 1 h at room temperature. Protein bands were visualized using SuperSignal™ West Pico PLUS Chemiluminescent Substrate (34580, Thermo Fisher Scientific, Waltham, MA, USA). Chemiluminescent signals were captured using an Amersham Imager 600 (GE Healthcare, Chicago, IL, USA), and the intensity of the bands was quantified using ImageJ software (version 1.52p; NIH, Bethesda, MD, USA).

### 4.6. Antibodies and Reagents

Rabbit primary antibodies were obtained from the following suppliers: Cell Signaling Technology (Danvers, MA, USA) [phospho-AMPK (2531), AMPK (2532), BDNF (47808)]; Bioss (Woburn, MA, USA) [ACTB (bs-0061R), CPT1A (bs-2047R), CPT2 (bs-5047R)]; Proteintech (Rosemont, IL, USA) [SIRT1 (13161-1-AP), PPARγ (16643-1-AP)]; Novus Biologicals (Centennial, CO, USA) [PGC-1α (NBP1-04676SS), PPARα (NB600-636SS)]; and Cusabio (Wuhan, Hubei, China) [NRF2 (CSB-PA003481)]. LPS (L2630) was purchased from Sigma-Aldrich (St. Louis, MO, USA).

### 4.7. Statistical Analysis

Data are presented as the mean ± SD. Statistical significance was evaluated using one- or two-way ANOVA, followed by the Holm–Sidak post hoc test for multiple comparisons. Comparisons between two groups were performed using Student’s *t*-test. All statistical analyses were conducted using SigmaPlot software (version 14.0; Systat Software, Inc., San Jose, CA, USA). A *p* value < 0.05 was considered statistically significant (* *p* < 0.05, ** *p* < 0.01, *** *p* < 0.001).

## Data Availability

The original contributions presented in this study are included in the article/Supplementary Materials. Further inquiries can be directed to the corresponding author.

## Supplementary Materials

The following supporting information can be downloaded at: https://www.mdpi.com/article/10.3390/plants15132056/s1, Figure S1: Original Western blot images of Figure 2; Figure S2: Original Western blot images for Figure 4.

## Abbreviations

AMPK, AMP-activated protein kinase; SIRT1, sirtuin 1; PGC-1α, peroxisome proliferator-activated receptor gamma coactivator 1-alpha; PPAR, Peroxisome Proliferator-Activated Receptor; CPT, carnitine palmitoyltransferase; NRF, nuclear factor erythroid 2-related factor 2; NO, nitric oxide; IL-6, interleukin-6; TNF-α, tumor necrosis factor-α; BDNF, brain-derived neurotrophic factor; DM, *Dendropanax morbifera*; YDM, young DM; ROS, reactive oxygen species; HPLC, high-performance liquid chromatography; DMEM, Dulbecco’s Modified Eagle Medium; FBS, fetal bovine serum; LPS, lipopolysaccharide; SD, standard deviation; ANOVA, analysis of variance.
