# Supplementary figures and images for: Comparative Study of Young and Mature Dendropanax morbifera Leaves: Superior Neuroprotective Efficacy of Young Leaves Through Enhanced Anti-Inflammatory and Metabolic Modulation
Source: Plants (Basel). 2026 Jul 2;15(13):2056. doi: 10.3390/plants15132056 (PMC13364310; doi:10.3390/plants15132056)

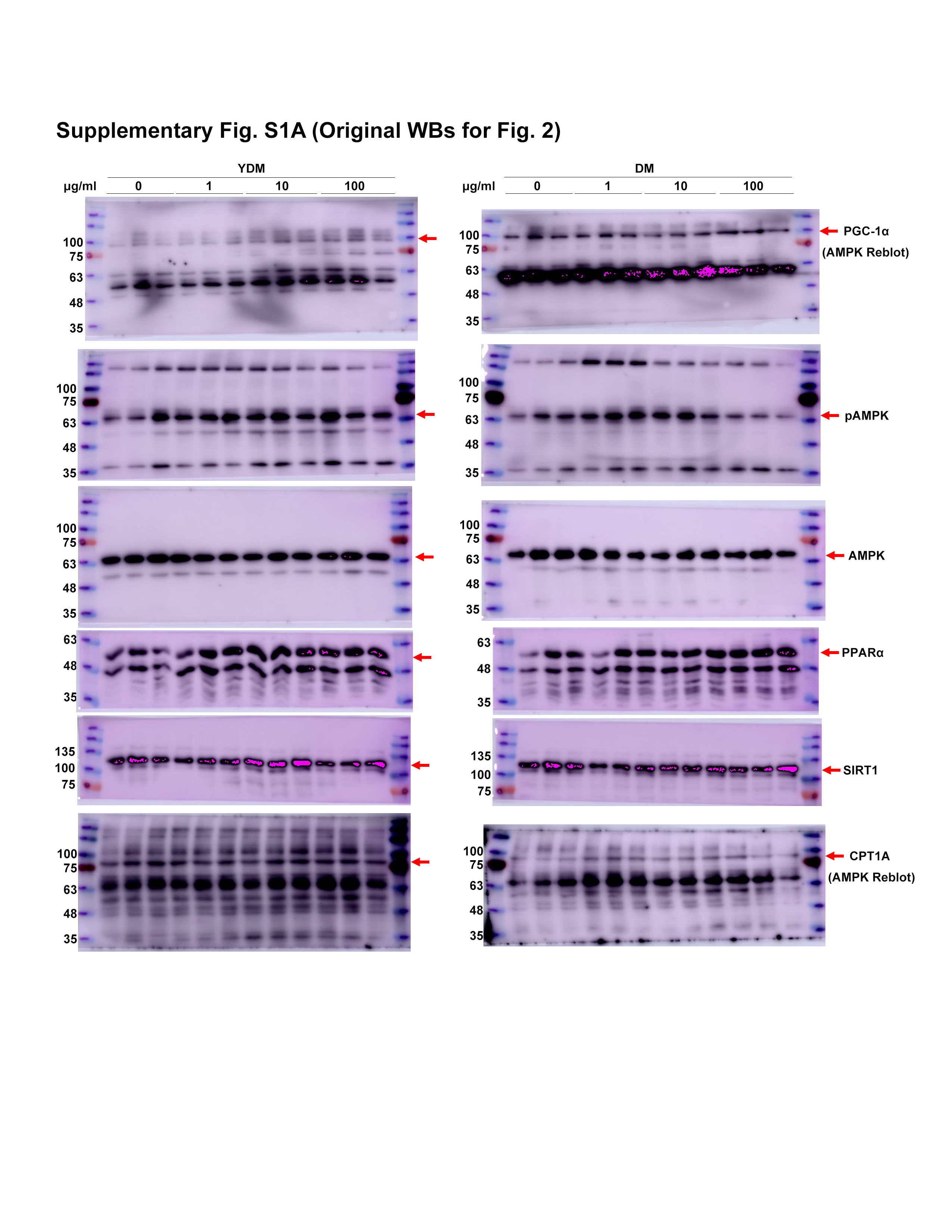

Supplement: Supplementary file 1 [file plants-15-02056-s001.zip › plants-4351419-Figure S1A (for Original WBs of Fig2).tif]

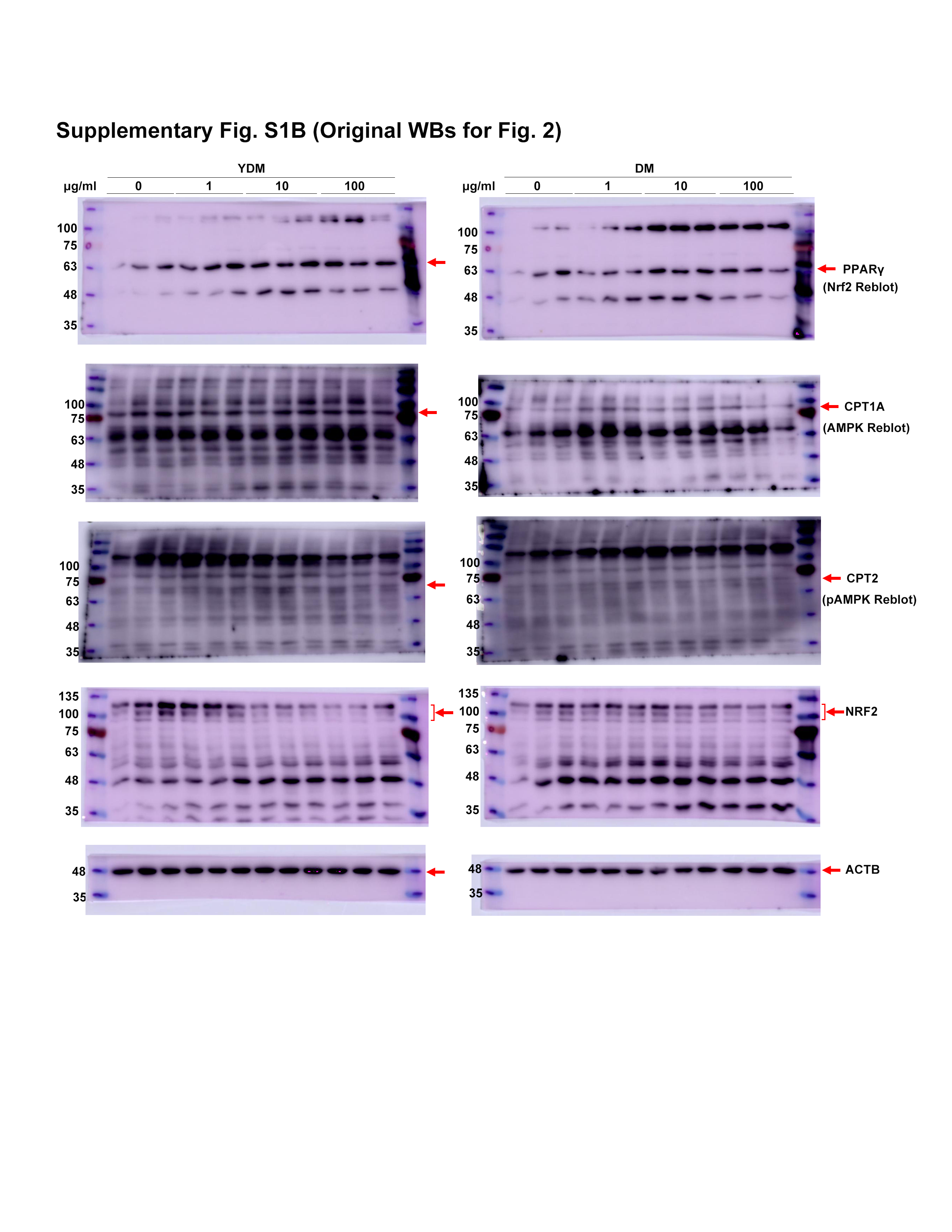

Supplement: Supplementary file 1 [file plants-15-02056-s001.zip › plants-4351419-Figure S1B (for Original WBs of Fig2).tif]

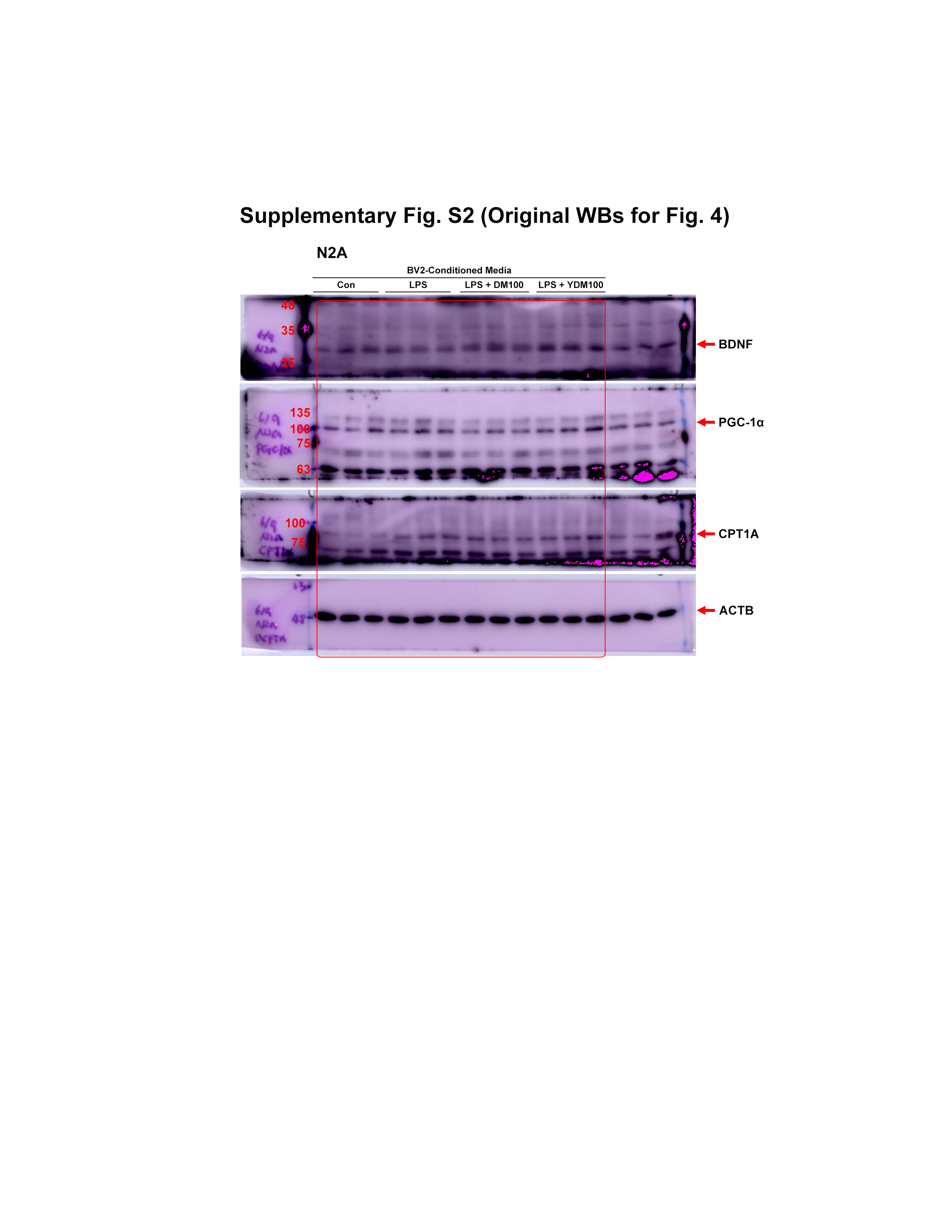

Supplement: Supplementary file 1 [file plants-15-02056-s001.zip › plants-4351419-Figure S2 (for Original WBs of Fig4).tif]
